# The Phenolic Antioxidant 3,5-dihydroxy-4-methoxybenzyl Alcohol (DHMBA) Prevents Enterocyte Cell Death under Oxygen-Dissolving Cold Conditions through Polyphyletic Antioxidant Actions

**DOI:** 10.3390/jcm10091972

**Published:** 2021-05-04

**Authors:** Moto Fukai, Takuya Nakayabu, Shintaro Ohtani, Kengo Shibata, Shingo Shimada, Soudai Sakamoto, Hirotoshi Fuda, Takayuki Furukawa, Mitsugu Watanabe, Shu-Ping Hui, Hitoshi Chiba, Tsuyoshi Shimamura, Akinobu Taketomi

**Affiliations:** 1 Department of Gastroenterological Surgery I, Graduate School of Medicine, Hokkaido University, Nishi 7, Kita 15, Kita-ku, Sapporo 060-8638, Hokkaido, Japan; nttakuya21@gmail.com (T.N.); ohtani.shintaro2l4@gmail.com (S.O.); kenshiba@pop.med.hokudai.ac.jp (K.S.); shimachine@hotmail.com (S.S.); soudai114@gmail.com (S.S.); taketomi@med.hokudai.ac.jp (A.T.); 2 Faculty of Health Sciences, Graduate School of Health Sciences, Hokkaido University, Nishi5, Kita12, Kita-ku, Sapporo 060-0812, Hokkaido, Japan; hirofuda@gmail.com (H.F.); s030247e@gmail.com (T.F.); gakujutsu@oyster.co.jp (M.W.); keino@hs.hokudai.ac.jp (S.-P.H.); chiba-h@sapporo-hokeniryou-u.ac.jp (H.C.); 3 Watanabe Oyster Laboratory Co. Ltd., 490-3, Shimoongata-cho, Hachioji 190-0154, Tokyo, Japan; 4 Department of Nutrition, Sapporo University of Health Sciences, 1-15, 2 chome, Nakanumanishi4jou, Higashi-ku, Sapporo 007-0894, Hokkaido, Japan; 5 Division of Organ Transplantation, Central Clinical Facilities, Hokkaido University Hospital, Nishi5 Kita14, Kita-ku, Sapporo 060-8648, Hokkaido, Japan; t_shima@med.hokudai.ac.jp

**Keywords:** enterocytes, IEC-6, cold preservation, hypoxia, oxidative stress, antioxidant, DHMBA, mitochondria, survival signal, lipid peroxidation

## Abstract

Cold preservation in University of Wisconsin (UW) solution is not enough to maintain the viability of the small intestine, due to the oxidative stress. The novel phenolic antioxidant 3,5-dihydroxy-4-methoxybenzyl alcohol (DHMBA) has dual properties to reduce oxidative stress, radical scavenging, and antioxidant protein induction, in other cells. This study was designed to determine whether DHMBA reduces cold preservation injury of enterocytes, and to identify the effector site. Enterocytes were subjected to 48-h cold preservation under atmosphere in UW solution (±DHMBA), and then returned to normal culture to replicate reperfusion of the small intestine after cold preservation. At the end of cold preservation (ECP) and at 1, 3, 6, and 72 h after rewarming (R1h, R3h, R6h, and R72h), we evaluated cell function and the injury mechanism. The results showed that DHMBA protected mitochondrial function mainly during cold preservation, and suppressed cell death after rewarming, as shown by the MTT, ATP, mitochondrial membrane potential, LDH, and lipid peroxidation assays, together with enhanced survival signals (PI3K, Akt, p70S6K) and induction of antioxidant proteins (HO-1, NQO-1, TRX-1). We found that DHMBA mitigates the cold-induced injury of enterocytes by protecting the mitochondria through direct and indirect antioxidative activities.

## 1. Introduction

In small intestinal transplantation, reperfusion of the small intestine after cold preservation impairs the absorption and barrier functions of the intestinal mucosa, leading to bacterial translocation, sepsis, re-transplantation, and mortality [1]. Rapid progression of the antigen-nonspecific innate immune response due to ischemia and reperfusion activates the initial response of acute rejection [2]. The safe time limit for cold preservation in UW solution is reported to be 8 h [3]. Since the standard cold preservation method is effective for other organs and is inefficient for the small intestine [3], the peculiarity of the small intestine should be considered. At the same time, protection of mitochondria and the cytoskeleton is also required [4].

The initial inflammatory reaction and cell death begin during cold preservation and peak at 6 h after reperfusion [5]. Necrotic, but not apoptotic, cell death promotes rejection [6]. Methods that supply oxygen to the graft at low temperatures, including the two-layer method [7] and intraluminal perfusion [8], have been useful in reducing necrosis. However, increased oxidative phosphorylation during cold preservation causes cell death associated with oxidative stress [9]. Supplementing the organ preservation solution with antioxidants reduces cold preservation and reperfusion-related injury of the small intestine [10]. However, antioxidants with different membrane permeabilities can induce different injury mitigation effects [11]. We hypothesized that an amphipathic antioxidant that can penetrate the cell membrane could reach the mitochondria in small intestinal epithelial cells exposed to the lumen. Such an antioxidant could reduce cold preservation injury by inhibiting oxidative stress resulting from mitochondrial respiration at low temperatures. However, given the lack of clinical application of many known antioxidants, there is a concern that the radical scavenging effect alone may be insufficient for clinical applications [10,12]. Therefore, we sought to investigate the protective effects of oxygen-dissolving cold conditions on the small intestine using 3,5-dihydroxy-4-methoxybenzyl alcohol (DHMBA).

DHMBA is a polyphenolic antioxidant extracted from the Pacific oyster (*Crassostrea gigas*) [13]. DHMBA is highly soluble in water, eliminates hydroxyl radicals, and inhibits hepatocyte DNA fragmentation and cell death [14]. In addition, DHMBA was shown to stimulate Nrf2 transcriptional activity, augmenting the expression of heme oxygenase-1 (HO-1), NAD(P)H quinone dehydrogenase 1 (NQO-1), superoxide dismutase 1 (SOD-1), and many other phase 2 enzymes [15]. Since the expression of antioxidant proteins is regulated partly by oxidative stress and redox status [16], it is unclear whether the antioxidant protein expression-inducing effect is exerted when there is a reduction in oxidative stress under oxygen-dissolving cold conditions. Thus, we constructed an in vitro experimental system that mimicked a small intestinal graft preserved under oxygen-dissolving cold conditions or through luminal oxygenated perfusion.

The purpose of this study was to determine whether DHMBA reduces cold preservation-induced injury under oxygen-dissolving cold conditions, whether it reduces oxidative stress and induces antioxidant protein expression, and the nature of these protective actions.

## 2. Materials and Methods

### 2.1. Chemicals and Reagents

All reagents were of the highest commercially available grade. DHMBA was synthesized, as previously described [17]. The 3-(4,5-dimethylthiazol-2-yl)-2,5-diphenyltetrazolium bromide (MTT) dye and the JC-1 fluorescent probe were purchased from Dojindo (Kumamoto, Japan). Antibodies against PI3K, phospho-PI3K Tyr^458^, Akt, phospho-Akt Ser^473^, mTOR, phospho-mTOR Ser^2448^, p70S6K, phospho-p70S6K Thr^389^, β-actin, apoptosis-inducing factor (AIF), COX IV, and goat anti-rabbit IgG antibody conjugated with horseradish peroxidase, were purchased from Cell Signaling Technology (Beverly, MA, USA). Antibodies against HO-1 and NQO-1 were purchased from Abcam (Cambridge, UK). Primary and secondary antibodies were diluted in tris-buffered saline (pH 7.4) with Tween20 (0.05%, *v*/*v*) to 1:1000 and 1:5000, respectively. Following reagents were purchased from Fuji Film Wako Pure Chemical Co. (Osaka, Japan): Trolox, Dimethyl sulfoxide (DMSO), Edaravone, Sodium fluoride, Sodium orthovanadate (V), and Bovine serum albumin fraction V (BSA). Following chemicals were purchased from Merck Inc. (Darmstadt, Germany): Deferoxamine mesylate (DFX), Ebselen, Protease inhibitor cocktail, and 2-Mercaputoethanol (2-ME).

### 2.2. Cell Culture

The small intestinal epithelial cell line (IEC-6; CRL1592), derived from rat, was purchased from the American Type Culture Collection (ATCC, Manassas, VA, USA) and cultured in Dulbecco’s Modified Eagle’s Medium supplemented with 10% (*v*/*v*) fetal bovine serum, penicillin (100 U/mL), and streptomycin (100 μg/mL) (Thermo Fisher Scientific, Waltham, MA, USA), according to the manufacturer’s instructions. For sampling, the cells were cultured to reach subconfluence on 10 cm dishes and subjected to cold preservation thereafter. Seeding densities for a 96-well plate (FluoroNunc and LumiNunc) and 16-well chamber slides (Nunc Lab-Tek chamber slide™) were 2 × 10^4^ and 1 × 10^5^ cells/well, respectively (Thermo Fisher Scientific, Waltham, MA, USA).

### 2.3. Cold Preservation and Subsequent Rewarming

The cells were preserved in ice-cold University of Wisconsin (UW) solution (10 mL) with or without test compounds, and then incubated at 4 °C for 48 h, mimicking the conditions of small intestinal epithelial cells of cold-preserved small intestine. Subsequently, the cells were returned to normal culture conditions in a CO2 incubator with the culture medium described above at 37 °C, mimicking the reperfusion phase in transplantation.

### 2.4. Experimental Protocol

#### 2.4.1. Protocol 1

The cytoprotective effects of DHMBA and known antioxidants were compared by cold preservation in UW solution (Belzar UW ®, Bridge to Life Co., Northbrook, IL, USA) at 4 °C for 48 h with these drugs or without (no treatment control; NT). Injury and viability were evaluated at the end of cold preservation (ECP) and after rewarming for 72 h (R72h). DHMBA (DH; 1 mM), DFX (100 µM), and Trolox (Trl; 100 µM) were directly dissolved in the UW solution. Edaravone (Edr) and Ebselen (Ebs) in DMSO (1000× stock) were added to UW solution at final concentrations of 1 mM (Edr) and 10 µM (Ebs), respectively. The optimal concentrations of all drugs were chosen by the dose response studies (Figures S1 and S2).

#### 2.4.2. Protocol 2

The cytoprotective effect of DHMBA was assessed. The acting point of the DHMBA was screened. Experimental groups were defined as no treatment (NT) and DHMBA (DH) groups.

### 2.5. Evaluations

The following assays were performed using a multimode microplate reader Varioskan Flash (Thermo Fisher Scientific).

#### 2.5.1. Viability and Injury

Viability was assessed by the MTT and ATP assays, as previously described [15] (*n* = 8 each). Briefly, the MTT assay was started at the indicated time points by incubating the cells with the culture medium containing MTT (0.5 mg/mL) at 37 °C for 2 h. Next, the absorbance at 570 nm was measured. ATP content was measured using a luciferin-luciferase assay kit, CellTiter-GloLuminescent Cell Viability Assay (Promega Japan, Tokyo, Japan), according to the manufacturer’s instructions. Before subjecting the plate to cold preservation, MTT assay was performed in each plate (8 wells/plate) as internal control of the assay; defined as “Normal cells”. All values are expressed as a percentage compared to the mean values in normal cells.

#### 2.5.2. Lactate Dehydrogenase (LDH) Assay

Cellular injury was evaluated based on LDH activity in the test solution or culture medium using the Takara Cytotoxicity Detection Kit™ (Takara Bio Inc., Shiga, Japan), according to the manufacturer’s instructions. To know the total cell number just before cold preservation, cells were collected in phosphate buffered saline with 0.2% Tween20 (PBS-T) by scraping and vigorous pipetting (8 wells/plate). Then, collected cells were lysed by freeze and thaw, and sonication thereafter. LDH activity in this lysate served as the internal control in each plate. All values are expressed as a percentage compared to the lysate before cold preservation, which indicates the dead cell ratio (%).

#### 2.5.3. Mitochondrial Membrane Potential

Mitochondrial membrane potential was evaluated using a JC-1 fluorescent probe (Dojindo, Kumamoto, Japan) and a microplate reader (*n* = 8). Briefly, the cells were subjected to cold preservation under an atmosphere in the UW solution, with or without DHMBA. Then, the preservation solution was exchanged to the normal culture medium containing JC-1 probe, and the cells were incubated for 15 min at 37 °C. After removing the extracellular dye, the fluorescence intensity was then measured at excitation (Ex.) and emission (Em.) wavelengths of 550 nm and 600 nm, respectively, to detect high membrane potential, while mitochondria with low membrane potential were measured at wavelengths of 485 (Ex.) and 535 (Em.) nm, respectively, using multimode microplate reader. The ratio of the mean fluorescence intensity (MFI), MFI_555/600_/MFI_485/535_, was calculated. Values are expressed as a percentage compared to that separately obtained in normal cells. Cells seeded on a 16-well chamber slide (Thermo Fisher Scientific) were observed and images were obtained using a fluorescent microscope (BZ-9000; Keyence, Osaka, Japan). Cells were presented as red (555/600; Ex./Em.) and green (485/535; Ex./Em.) pseudocolors.

#### 2.5.4. Lipid Peroxidation Assay

The summation of malondialdehyde (MDA) and 4-hydroxy-2-nonenal (4-HNE) was measured using the Bioxytech LPO-586 kit (Oxis Health Product Inc., Portland, OR, USA), in accordance with the manufacturer’s instructions. Values are expressed as nmol MDA equivalent per mg protein, using the MDA standard for the standard curve. Protein concentration was measured using a BCA protein assay kit (Thermo Fisher Scientific).

#### 2.5.5. Cell Survival and Death

Cell survival and cause of death were evaluated using an Apoptotic/Necrotic/Healthy Cells Detection Kit (Takara Bio Inc., Shiga, Japan), in accordance with the manufacturer’s instructions. Briefly, the cells cultured on a 16-well chamber slide (Thermo Fisher Scientific) were subjected to cold preservation and subsequent rewarming for 3 h (R3h), and then stained with Hoechst33324 (350/461 nm), FITC-conjugated Annexin V (492/514 nm), and ethidium homodimer III (EthD III; 528/617 nm); excitation and emission wavelengths were described as Ex/Em nm. Images were obtained using a fluorescence microscope (BZ-9000; Keyence). After fixing the optimal conditions for each dye, cells were imaged and counted thereafter using the BZ-II dynamic cell count software (Keyence, Osaka, Japan).

#### 2.5.6. Western Blotting

Samples were collected from cold-preserved cells at the ECP, R1h, R3h, and R6h, and from normal cells. Twelve dishes at each timepoint were collected into 3 separate samples (*n* = 3). Cells were lysed with HEPES-sucrose buffer (10 mM, pH 7.5) containing a protease inhibitor cocktail, sodium fluoride (5 mM), and sodium orthovanadate (5 mM). After serial centrifugation of 1000× *g* for 10 min, 20,000× *g* for 25 min, and 20,000× *g* for 25 min again, cytoplasmic and mitochondrial fractions were collected. The protein concentration was measured using a BCA Protein Assay Kit (Thermo Fisher Scientific).

The protein extracts were denatured by boiling in a sample buffer (6X) for sodium dodecyl sulfate-polyacrylamide gel electrophoresis (SDS-PAGE) (Nacalai TESQUE Inc., Kyoto, Japan) and 2-mercaptoethanol. Subsequently, the samples (12 µg protein) were applied to an Any-kD Pre-cast Gel (Bio-Rad, Hercules, CA, USA) and subjected to standard SDS-PAGE. After protein transfer to a membrane, it was blocked with 3% (*w/v*) BSA, incubated with primary and secondary antibodies, and immersed in the West Dura chemiluminescence detection reagent (Thermo Fisher Scientific). Band densities were measured using a chemiluminescence detector (Chem Doc XRS™; Bio-Rad), and cytoplasmic and mitochondrial proteins were normalized to β-actin and COX IV, respectively. These values were finally converted to percentages versus the values obtained from normal cells.

### 2.6. Statistics

**Figure 1:**
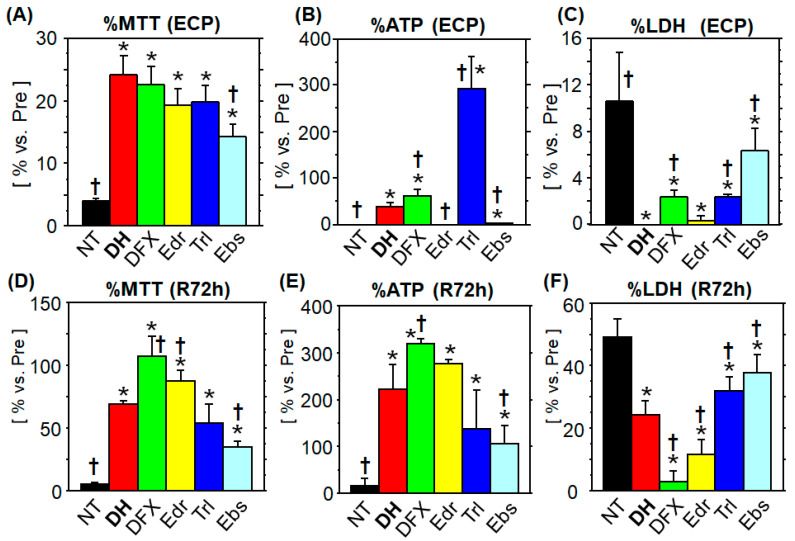
Cytoprotective ability of different antioxidants during cold preservation and subsequent rewarming. Enterocytes were subjected to 48-h cold preservation (CP) in UW solution (no treatment; NT) or with DHMBA (DH), Deferoxamine (DFX), Edaravone (Edr), Trolox (Trl), or Ebselen (Ebs). Mitochondrial function was assessed by MTT assay and ATP content. Values are expressed as a percentage versus the mean value in normal cells before CP. Cellular injury was evaluated by measuring LDH activity in the test solution or culture medium. Values are expressed as a percentage compared to the cell lysate obtained before CP. (**A**–**C**): at the end of cold preservation (ECP); (**D**–**F**): after rewarming for 72 h (R72h). All treatment groups showed significantly higher values in the MTT assay and significantly lower values of LDH leakage compared to those of the NT group at the ECP and R72h. ATP content at the ECP was almost undetectable in the Edr, Ebs, and NT groups, whereas it was significantly higher at R72h in all the treatment groups than in the NT group. (*n* = 8, mean ± SD, * *p* = 0.0085 vs. NT group, Steel’s test; † *p* ≤ 0.0171 vs. DH group, Steel-Dwass test).

**Figure 2:**
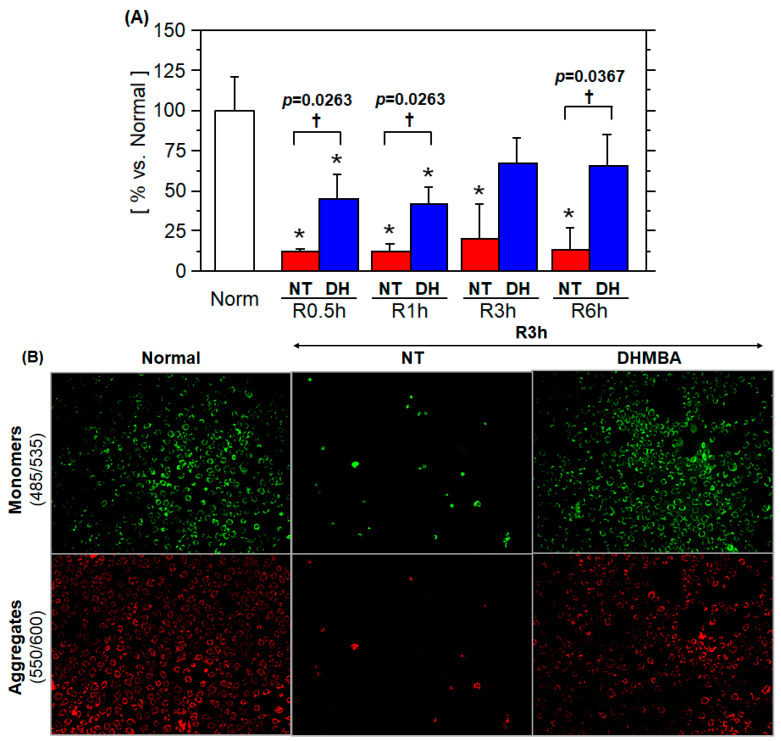
Mitochondrial membrane potential (ΔΨ). Enterocytes were subjected to 48 h cold preservation (CP) in UW solution without antioxidant (no treatment; NT) or with DHMBA (DH; 1 mM). The ΔΨ probe JC-1 was loaded immediately at rewarming, and the fluorescent intensity was measured at excitation and emission wave lengths of 550 and 600 nm for aggregated dye (high ΔΨ), and 485 and 535 nm for monomers (low ΔΨ) up to 6 h. The ratio of the mean fluorescent intensity (MFI), MFI_555/600_/MFI_485/535_, was calculated and normalized against the ratio in normal cells (*n* = 8, mean ± SD, * *p* ≤ 0.0142 vs. Normal, Steel’s test; † *p* ≤ 0.0367, NT vs. DH groups, Steel-Dwass test). (**A**) The ΔΨ of the DH group was significantly higher than that of the NT group throughout the experiment. (**B**) Representative images at R3h are shown.

**Figure 3:**
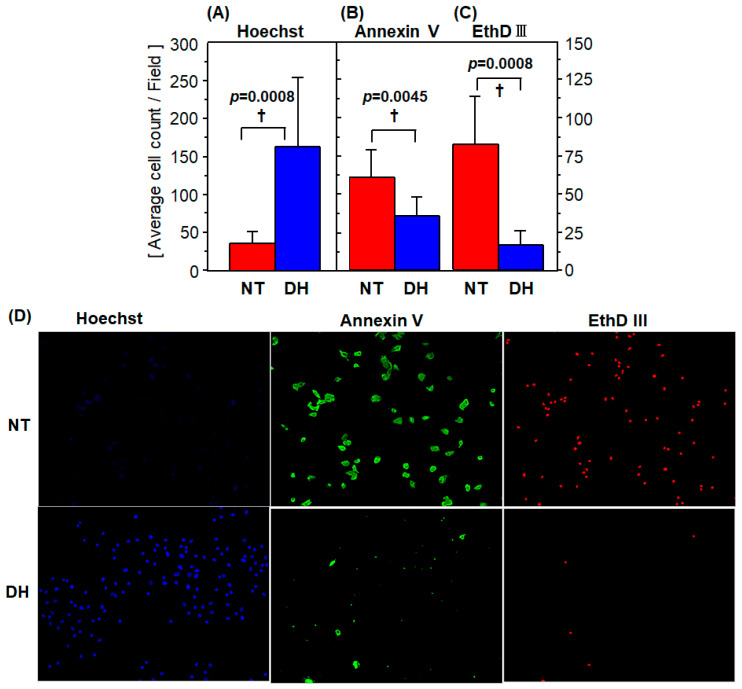
Fluorescent imaging to assess cell death and survival. Enterocytes were subjected to a 48 h cold preservation under atmospheric conditions in UW solution with or without DHMBA (1 mM) and subsequent normal culture for 3 h (R3h). Then cells were stained with Hoechst33342, Annexin V, and Ethidium homodimer III to assess cell (**A**) survival, (**B**) apoptosis, and (**C**) necrosis, presented in pseudo-coloring of blue, green, and red, respectively. Positively stained cells for the 3 dyes were counted (*n* = 8). (**A**) Live cell count was significantly increased in the DH group. (**B**,**C**) Apoptosis and necrosis were significantly reduced in the DH group (*n* = 8, mean ± SD, † *p* ≤ 0.0045, NT vs. DH group, Mann–Whitney’s U test). **(D)** Representative images at R3h are shown.

**Figure 4:**
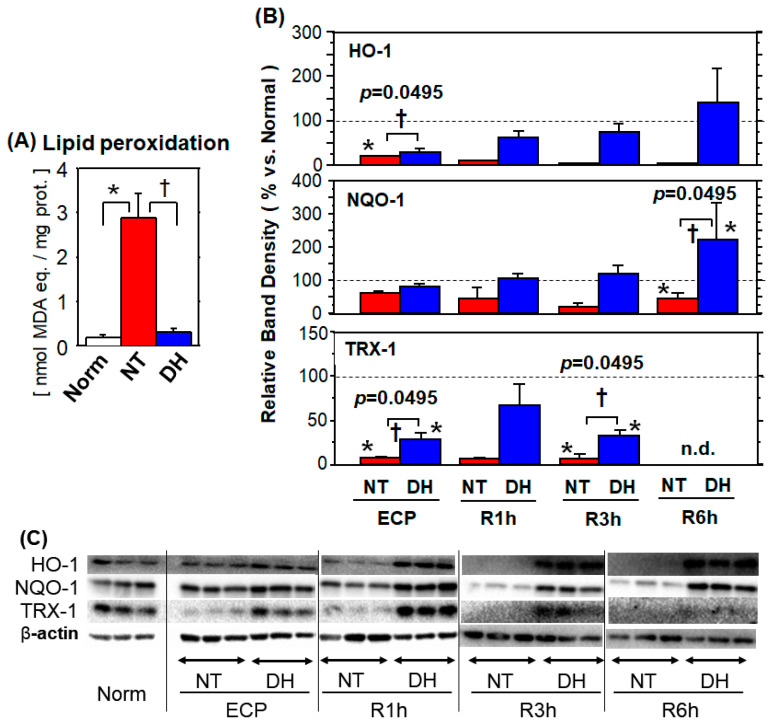
Oxidative stress and antioxidant enzymes. Enterocytes were subjected to 48 h cold preservation (CP) in UW solution without antioxidant (no treatment; NT) or with DHMBA (DH) and subsequent normal culture (rewarming) for up to 6 h. (**A**) Lipid peroxidation (LPO). LPO product measured by LPO586 kit at R1h was significantly higher in the NT group (vs. normal cells), whereas in the DH group the increase was significantly suppressed. (*n* = 4; mean ± SD, * *p* < 0.05, vs. normal, Dunn’s test; † *p* < 0.05, NT vs. DH groups, Mann–Whitney U-test) (**B**) Antioxidant enzymes. HO-1, NQO-1, and TRX-1 levels were determined by western blotting up to R6h. Band density was standardized by beta-actin and by the average value in normal cells. In the NT group, the level of these proteins was significantly lower (vs. normal), whereas in the DH group, HO-1 and NQO-1 showed higher value. Although TRX-1 showed significantly higher value in the DH group up to R3h, TRX-1 levels were still lower than those of normal cells. (*n* = 3; mean ± SD, * *p* < 0.05, vs. normal; † *p* < 0.05, NT vs. DH groups, Mann–Whitney U-test). (**C**) Representative blots are shown.

**Figure 5:**
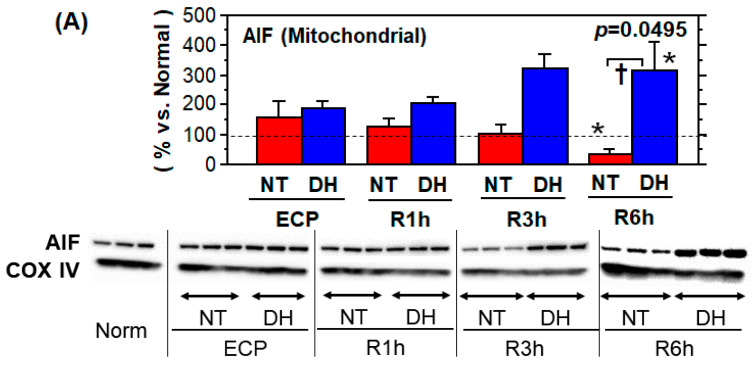

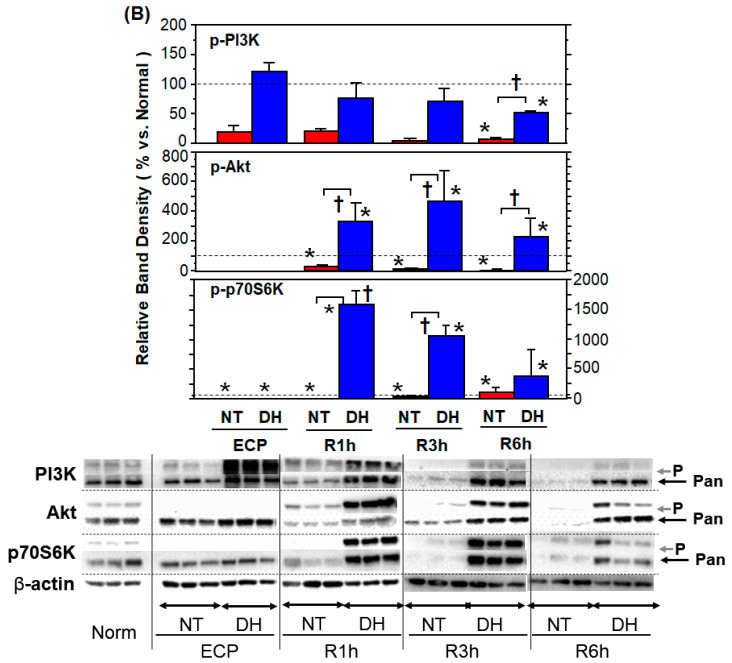
Cell death and survival signals. Enterocytes were subjected to 48 h cold preservation (CP) in UW solution (no treatment; NT) or with DHMBA (DH). Subsequently, the cells were normally cultured for up to 6 h and applied to western blotting. Band density was normalized by COX IV for mitochondrial AIF and by beta-actin for others, and further standardized by the average values in normal cells (*n* = 3; mean ± SD, * *p* < 0.05, vs. normal; † *p* = 0.0495, NT vs. DH groups, Mann–Whitney U-test). (**A**) Mitochondrial AIF levels decreased from R1h to R6h in the NT group and increased in the DH group. (**B**) In the NT group, a decrease in expression of the phosphorylated forms of PI3K (p-PI3K), Akt (p-Akt), and p70S6K (p-p70S6K) was observed. In the DH group, p-PI3K levels were almost unchanged throughout the experiment. p-Akt and p-p70S6K levels remained notably greater than the normal level up to R6h.

Data are expressed as mean ± standard deviation (SD). The difference between groups of interest is statistically evaluated by JMP^®^ Pro 14.0.0 (SAS Institute Inc., Cary, NC, USA) software. For Figure 1 and Figure 2, Steel-Dwass and Steel’s tests are applied for all groups and for comparison to NT group, respectively. For Figure 3, Figure 4 and Figure 5, Mann–Whitney’s U tests are applied to NT and DH groups after confirming the applicability by Kruskal–Wallis test for 3 groups comparison (Norm, NT, and DH groups).

## 3. Results

### 3.1. Comparison of the Cytoprotective Ability

#### 3.1.1. MTT Assay

At the ECP, the NT group showed a significant decrease in MTT metabolism (4.0% ± 0.3% vs. normal), whereas all the treatment groups showed significantly increased MTT metabolism, compared to the NT group. Among the treatment groups, the Ebs group showed the lowest MTT metabolism, and the others showed an equivalent effect on MTT catabolism. Notably, the mean value for MTT metabolism was the highest in the DH group (24.2% ± 3.1% vs. normal; Figure 1A). At R72h, the NT group showed the lowest value (5.0% ± 1.2% vs. normal), and all treatment groups showed significantly higher MTT metabolism compared to the NT group. The MTT metabolism in the DH group (68.7% ± 3.0% vs. normal) was the third highest among the five tested agents. The DFX and Edr groups showed higher MTT metabolism, and the Trl and Ebs groups showed lower values than those of the DH group (Figure 1D).

#### 3.1.2. ATP Content

At the ECP, ATP was almost undetectable in the NT and Edr groups (0.86% ± 0.14%, 0.93% ± 0.15%, respectively, vs. normal) and was significantly higher in the other treatment groups. In the DH group, ATP levels were significantly lower (24.2% ± 3.1% vs. normal) than those in the Trl and DFX groups, and significantly higher than those in the Ebs group (Figure 1B). The lowest ATP level (16.2% ± 14.6% vs. normal) was found at R72h in NT group, while ATP was significantly higher in the other treatment groups. 

#### 3.1.3. LDH Leakage

At the ECP, the NT group showed the lowest LDH activity (10.5% ± 4.2% vs. lysate of normal cells). The LDH activity in the test solution was undetectable in the DH and Edr groups, indicating strong cytoprotection. Among the treatment groups, the Ebs group showed the highest LDH activity (6.3% ± 2.0% vs. lysate of normal cells), whereas the DFX and Trl groups showed a significantly lower LDH activity (Figure 1C). At R72h, the NT group showed the highest LDH activity (49.1% ± 5.5% vs. lysate of normal cells). LDH activity was higher in the DFX and Edr groups and lower in the Trl and Ebs groups, as compared to that in the DH group (Figure 1F).

### 3.2. Mitochondrial Membrane Potential

Throughout the experiment, the mitochondrial membrane potential was significantly lower in the NT group than in the DH group. The mitochondrial membrane potential in the DH group was already increased to 45.6% ± 14.6% (vs. normal) at R0.5h, followed by a slight increase at R1h (41.6% ± 11% vs. normal), R3h (67.4% ± 15.4% vs. normal), and finally reaching 65.5% ± 19.3% (vs. normal) at R6h († *p* ≤ 0.0367, NT vs. DH groups; Figure 2A). Microscopy revealed that the number of cells was greater in the DH group than in the NT group at R3h (Figure 2B).

### 3.3. Cell Survival and Death

The number of the Hoechst33324-positive cells was 35.9 ± 16.3 cells/field in the NT group, and it increased significantly in the DH group to 162 ± 92 cells/field (Figure 3A). The number of Annexin V-positive cells was 60.3 ± 18.1 cells/field in the NT group, compared to 35.3 ± 12.2 cells/field in the DH group (Figure 3B). The number of the EthD-III-positive cells was 82.4 ± 30.9 cells/field in the NT group; this number decreased significantly in the DH group to 16.5 ± 9.6 cells/field (Figure 3A). DHMBA treatment resulted in a 42% and 80% reduction in apoptotic and necrotic cells, respectively († *p* ≤ 0.0045, NT vs. DH groups; Figure 3A–C). 

More detailed observations using fluorescence microscopy revealed that Hoechst33324- and EthD III-positive cells, which both clearly stained in the nucleus, could be easily determined by eye as well as by BZ-II automatic cell count software (Keyence). In contrast, Annexin V-positive cells were difficult to detect automatically because of the lack of homogeneity, partial staining in the cells, and the presence of cellular fragments. Additionally, it was sometimes difficult to distinguish cellular fragments from contracted cells (Figure 3D).

### 3.4. Oxidative Stress

#### 3.4.1. Lipid Peroxidation

At R1h, the lipid peroxidation product was 15.9 times higher in the NT group compared to that in normal cells. This increase was significantly reduced to 1.7 times (vs. normal) in the DH group (* *p* < 0.05, vs. normal; † *p* < 0.05, NT vs. DH groups; Figure 4A). 

#### 3.4.2. Antioxidant Enzymes

Cytoplasmic HO-1 protein expression was significantly lower in the NT group (vs. normal) throughout the experiment. In the DH group, HO-1 expression increased over time and was significantly higher, compared to that in the NT group, from R1h to R6h.

Cytoplasmic NQO-1 protein expression was below the normal level throughout the experiment and was significantly lower (vs. normal) at R3h and R6h. In the DH group, cytoplasmic NQO-1 protein expression decreased to 82.1% ± 7.1% (vs. normal) at the ECP, while it increased to 106% ± 14% (vs. normal) and 223 ± 108% (vs. normal) at R1h and R6h, respectively. Notably, in the DH group NQO-1 protein expression was significantly higher than in the NT group throughout the experiment.

The expression profile of TRX-1 differed from that of HO-1 and NQO-1. In the NT group, cytoplasmic TRX-1 protein expression was significantly lower throughout the experiment. In the DH group, although it was the highest at R1h (67.4% ± 24.6% vs. normal) and decreased thereafter, the values from the ECP to R3h were significantly higher than those in the NT group. At R6h, TRX-1 was not detected in both the NT and DH groups (* *p* < 0.05, vs. normal; † *p* = 0.0495, NT vs. DH groups; Figure 4B).

Cytoplasmic HO-1 protein expression was significantly lower in the NT group (vs. normal) throughout the experiment. In the DH group, HO-1 expression increased over time and was significantly higher, compared to that in the NT group, from R1h to R6h. Cytoplasmic NQO-1 protein expression was below the normal level throughout the experiment and was significantly lower (vs. normal) at R3h and R6h. In the DH group, cytoplasmic NQO-1 protein expression decreased to 82.1% ± 7.1% (vs. normal) at the ECP, while it increased to 106% ± 14% (vs. normal) and 223 ± 108% (vs. normal) at R1h and R6h, respectively. Notably, in the DH group NQO-1 protein expression was significantly higher than in the NT group throughout the experiment. Cytoplasmic HO-1 protein expression was significantly lower in the NT group (vs. normal) throughout the experiment. In the DH group, HO-1 expression increased over time and was significantly higher, compared to that in the NT group, from R1h to R6h.

The expression profile of TRX-1 differed from that of HO-1 and NQO-1. In the NT group, cytoplasmic TRX-1 protein expression was significantly lower throughout the experiment. In the DH group, although it was the highest at R1h (67.4% ± 24.6% vs. normal) and decreased thereafter, the values from the ECP to R3h were significantly higher than those in the NT group. At R6h, TRX-1 was not detected in both the NT and DH groups (* *p* < 0.05, vs. normal; † *p* = 0.0495, NT vs. DH groups; Figure 4B).

### 3.5. Stress Responses and Survival Signals

The mitochondrial AIF level at the ECP tended to decrease after rewarming, with time, and reached 32.6% ± 19.7% (vs. normal) at R6h. Conversely, in the DH group, mitochondrial AIF tended to increase with time, and reached to 312 ± 96.8% (vs. normal) at R6h. (* *p* < 0.05, vs. normal; † *p* = 0.0495, NT vs. DH groups; Figure 5A).

The phosphorylated PI3K (Tyr^458^) (p-PI3K) level in the NT group peaked at R1h (21.0% ± 4.4% vs. normal) and maintained low values thereafter. In the DH group, p-PI3K (Tyr^458^) level was the highest at the ECP (122% ± 15.2% vs. normal) and gradually decreased thereafter, reaching 50.6% ± 3.6% (vs. normal) at R6h. (* *p* < 0.05, vs. normal; † *p* = 0.0495, NT vs. DH groups; Figure 5B).

The phosphorylated Akt (Ser^473^) (p-Akt) level in the NT group peaked at R1h (28.8% ± 9.4% vs. normal) and being undetectable (<8.6% vs. normal) at R3h and R6h. Conversely, p-Akt level in the DH group was undetectable at the ECP; however, phosphorylation exceeded the normal level at R1h (335% ± 122% vs. normal), R3h (469% ± 203% vs. normal), and R6h (229% ± 122% vs. normal), showing a significant augmentation, compared to the NT group. (* *p* < 0.05, vs. normal; † *p* = 0.0495, NT vs. DH groups; Figure 5B).

The phosphorylated of p70S6K (Thr^389^) (p-p70S6K) level was undetectable at the ECP and at R1h and increased to 29.2% ± 25.8% (vs. normal) at R3h and finally returned to 112% ± 79% (vs. normal) at R6h. In the DH group, p-p70S6K level was undetectable at the ECP. Notably, p-p70S6K increased dramatically to 1593 ± 225% (vs. normal) at R1h, 1063 ± 178% (vs. normal) at R3h, and 388 ± 444% (vs. normal) at R6h. (* *p* < 0.05, vs. normal; † *p* = 0.0495, NT vs. DH groups; Figure 5B).

## 4. Discussion

Organ perfusion of various organs has attracted increased attention, and the advantages and disadvantages of oxidative phosphorylation at low temperatures remains elusive [18]. In this study, we tested the cytoprotective effect of DHMBA in the cellular environment of small intestinal graft with dissolved oxygen, mimicking luminal perfusion [8] and the two-layer method [7]. DHMBA exhibited the greatest cytoprotective effect in the LDH and MTT assays at the ECP, indicating that it is superior to the clinically relevant antioxidants. However, DFX offered the most cytoprotection during rewarming, followed by Edr and DH (Figure S3). In addition, DHMBA is suitable for organ preservation and perfusion due to its attractive characteristics, such as amphipathic, induction ability of phase 2 enzymes, and water-soluble [15]. Edaravone for injection is dissolved in an acidic solution with a stabilizer, but it is extremely unstable at physiological pH [19]. Ebselen is dissolved in DMSO, which precipitates in aqueous phase due to its water-insolubility and freezing point of the solvent. DHMBA is free from these issues.

DHMBA treatment showed 41% reduction in apoptosis and 80% reduction in necrosis (Figure 3). Since cellular content leaked from dead cells, especially mitochondrial content, exacerbates ischemia and reperfusion injury [20], we assessed remnant dead cell number per live cell number as a risk of danger associated molecular patterns (DAMPs) mediated local injury. DHMBA treatment showed 86% reduction in apoptotic index and 95% reduction in necrotic index at R3h (Figure S3). Furthermore, the levels of accumulated LDH during the 72 h rewarming period was normalized by the viable cell number at the start of rewarming (%MTT at ECP). The index indicated that all antioxidant treatment during cold preservation remarkably inhibited post-rewarming cell death (Figure S4). Altogether, DHMBA reduced cell death during cold preservation and thereby protecting cells at the early phase of rewarming-reperfusion.

It is known that mitochondrial membrane potential, the electron transport chain, ATP synthesis, and reactive oxygen species (ROS) production and elimination do not have a uniform level of sensitivity to temperature changes [18]. In addition, oxygen concentration changes can cause an increase in ROS production [18]. ROS production and ATP depletion lead to intracellular Ca^2+^ overload and mitochondrial permeability transition pore opening, resulting in apoptosis and necrosis [21]. In this study, we observed higher mitochondrial membrane potential in the DHMBA group starting from 0.5 h after rewarming. It is of note that the higher %MTT at the ECP reflects not only the mitochondrial status only at the ECP but also drug metabolism from the ECP to the first 2 h of rewarming. Altogether, these results suggest that DHMBA protected the mitochondria during cold preservation, thereby enabling rapid recovery of mitochondrial function after rewarming, leading to reduced apoptosis and necrosis; however, the subsequent fate may be decided by other factors [21,22].

When lipid hydroperoxide is broken down to MDA and/or 4-HNE [23], it forms adducts with proteins, resulting in impairment of function [24]. Since DHMBA is amphipathic [13,17], it can be present in all subcellular fractions and function properly. Moreover, computational studies have revealed that DHMBA scavenges hydroperoxyl radicals (HOO·) with a rate constant higher than that of Trolox in aqueous and lipid phases, possibly chelates copper ions, and can self-recover this scavenging ability at physiological pH [25]. Thus, DHMBA has multiple potential functions, such as radical scavenging, as shown in Edaravone [26], Trolox [10], and Ebselen [27], and metal chelation, as shown in Deferoxamine [9]. Altogether, DHMBA appeared to protect enterocytes from cold preservation and rewarming injury through mitochondrial protection and radical scavenging, the “direct antioxidant actions” [15].

Next, we evaluated HO-1, NQO-1, and TRX-1 to verify whether DHMBA has “the indirect antioxidant actions” and the Nrf2-activating effect reported in other pro-electronic substances such as Butylated hydroxytoluene (BHT) [14], Curcumin, Sulforaphane [15], and Chlorogenic acid [28]. These proteins were more highly expressed in the DH group than in the NT group at R1h and R3h. Liang et al. reported that 4-HNE induced the upregulation of pro-apoptotic proteins, reduced the expression of tight junction proteins, and reduced the levels of nuclear Nrf2, resulting in a decrease in HO-1 and NQO-1 levels in porcine enterocytes [29]. In agreement with these reports, we confirmed the DHMBA-mediated upregulation of HO-1, NQO-1, and TRX-1. However, the time course of HO-1, NQO-1, and TRX-1 differed, suggesting different regulatory mechanisms. NQO-1 has both ARE and AP-1 sequences [30], and the HO-1 gene is positively regulated by Nrf2 and negatively regulated by NF kappa B [31]. Notably, these proteins were downregulated in the NT group at all time points, and this decrease was inhibited in the DHMBA group. Therefore, *de novo* protein expression, translocation from the cytoplasm to the nucleus and other fractions [32], and protein decomposition [33] should all be ruled out. In fact, translocation of HO-1 into the nucleus enhances the transcriptional activity of AP-1 [32] and Nrf2 [33], and translocation of TRX-1 into the nucleus regulates the transcriptional activity of HIF1α [34]. In addition, translocation of TRX-1 into the nucleus occurs when the intramolecular thiol is oxidized by hypoxia and reoxygenation [35]. Overall, the molecular mechanisms of DHMBA-mediated interaction, regulation, and translocation of nuclear proteins, including transcription factors and their products (phase 2 proteins), remain largely unknown and require further investigation.

During ischemia and reperfusion of the small intestine, PI3K-Akt [36] and mTOR-p70S6K activities [37] reduce tissue damage and inflammation. Additionally, the activation of PDK1-Akt-mTOR-p70S6K reduces injury during ischemia and reperfusion of the liver [38]. In this study, the levels of phosphorylated PI3K, Akt, mTOR, and p70S6K were low from the ECP to R6h in the NT group. However, PI3K phosphorylation was maintained at a normal level in the DH group, and phosphorylation of the other three proteins was maintained at higher than normal levels, resulting in the mitigation of both apoptosis and necrosis. Mitochondrial release of AIF induces apoptosis [39], whereas the translocation of AIF from the mitochondria to the nucleus is independent of ATP and caspase. 

Finally, the limitations of the present study should be disclosed. Protective effects of DHMBA on various cell types responsible for absorptive, secretory, and barrier functions as well as on mature, immature, and stem cells remain unknown. The biological functions of these cells and the ratio of different cell lineages are fundamental for maintaining homeostasis and promoting injury recovery [40]. Furthermore, drug metabolism, pharmacokinetics, interactions with blood cells, and morphology of villi and crypts are important issues to consider [8], which require further animal experiments.

## 5. Conclusions

DHMBA reduces cell death of small intestinal epithelium due to cold preservation under oxygen dissolved conditions through protection of mitochondria during cold preservation and mitigates both apoptosis and necrosis by direct antioxidant effect, and subsequent indirect action (antioxidant protein expression).

## Data Availability

The data presented in this study are available on request from the corresponding author..

## Supplementary Materials

The following are available online at https://www.mdpi.com/article/10.3390/jcm10091972/s1, Figure S1: Dose response of DHMBA, Figure S2: Dose responses of the known antioxidants, Figure S3: Apoptotic and necrotic cells numbers per live cell number, Figure S4: Cell death index during 72 h of rewarming.
