# Supplementary material for: The Phenolic Antioxidant 3,5-dihydroxy-4-methoxybenzyl Alcohol (DHMBA) Prevents Enterocyte Cell Death under Oxygen-Dissolving Cold Conditions through Polyphyletic Antioxidant Actions
Source: J Clin Med. 2021 May 4;10(9):1972. doi: 10.3390/jcm10091972 (PMC8124816; doi:10.3390/jcm10091972)
Supplement: Supplementary file 1 [file jcm-10-01972-s001.zip › jcm-1181804-supplementary.pdf]

Figure S1: Dose response of DHMBA. The optimal concentration of DHMBA was assessed by the LDH leakage at the end of 48-hour cold preservation (ECP) in UW solution as described in the *Materials and Methods* section with various concentrations of DHMBA. Values were expressed as % vs. NT group (n=8; Mean±SD). LDH leakage was the lowest at 1.0 mM at the ECP.

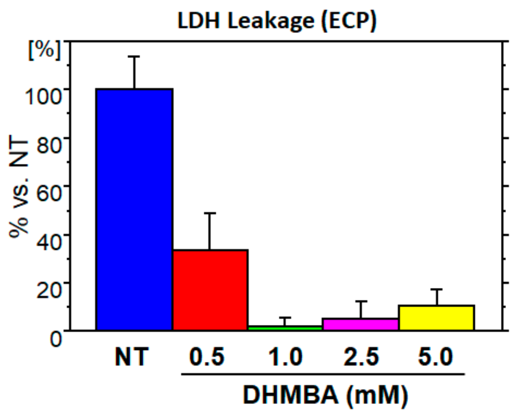

Figure S2: Dose responses of the known antioxidants. The optimal concentrations were evaluated by 48-hour cold preservation in UW solution with various concentrations of antioxidants, and subsequent MTT assay at ECP. Values were expressed as % vs. cells without cold preservation on the same plate (n=8; Mean±SD). The optimal concentrations appeared to be as follows: Edaravone (1mM), Deferoxamine (0.1mM), Ebselen(0.01mM), and Trolox (0.1mM).

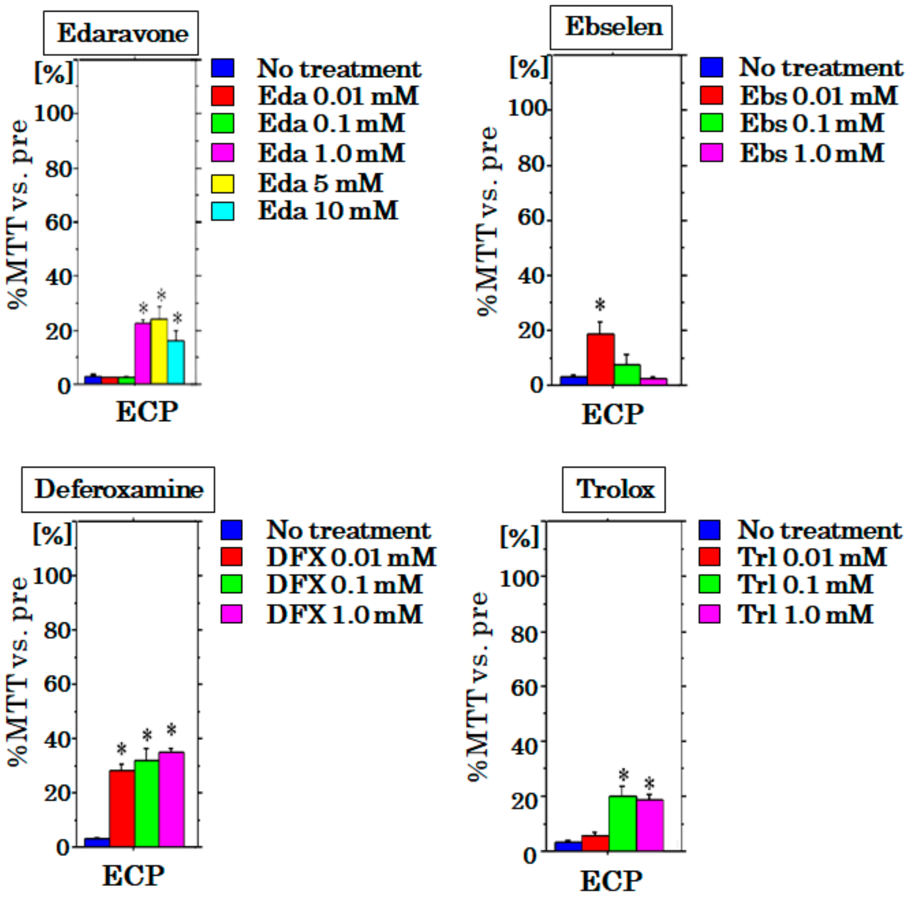

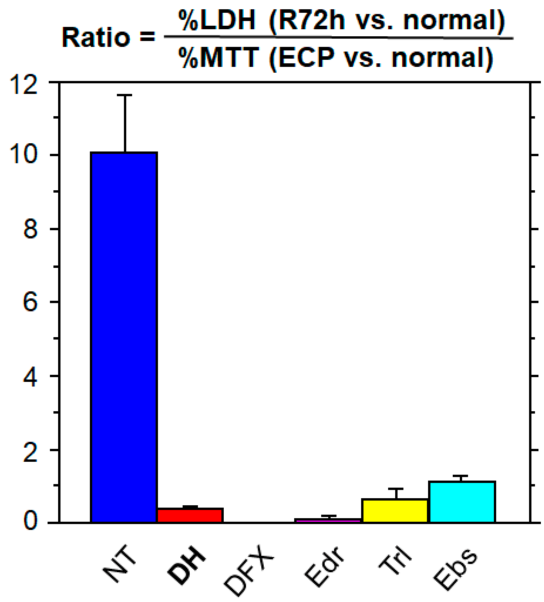

Figure S3: Cell death index during 72 h of rewarming. LDH leakage during 72 h from the end of cold preservation (ECP) was standardized by the viability determined using the MTT assay at the ECP; the cell death per residual viable cells is presented. The index revealed that antioxidant treatment during cold preservation prevented the progressive cell death after rewarming observed in the NT group. Protective ability of DHMBA after rewarming appeared to be comparable to that observed in clinically used drugs, edaravone and deferoxamine.

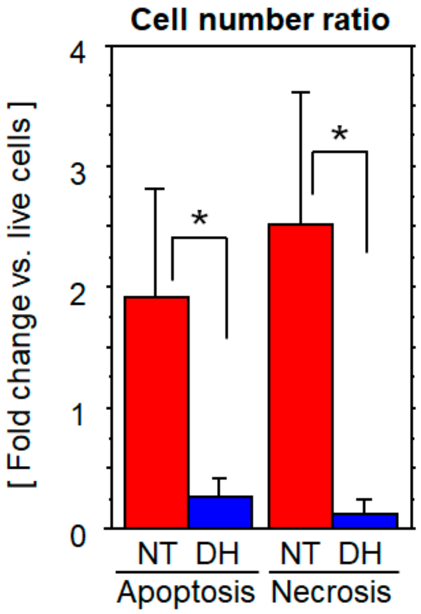

Figure S4: Apoptotic and necrotic cells numbers per live cell number. Dead but not detached cells are possible source of DAMPs. In other words, dead cells injure adjacent live cells. Accordingly, dead vs. alive cell number ratio (index) was determined. The index revealed that DHMBA reduced both apoptosis and necrosis. The DHMBA-mediated reduction rate of necrosis was higher than that of apoptosis (n=8; Mean±SD).
